# Dual mediating roles of friend support and self-esteem in the association between bicultural acceptance attitudes and life satisfaction among multicultural adolescents in South Korea: the moderating role of depression

**DOI:** 10.3389/fpsyg.2026.1817350

**Published:** 2026-05-11

**Authors:** Hai Lan Jin, Hua Mei Liu

**Affiliations:** 1 Department of Global Culture Contents, Hanseo University, Seosan City, Republic of Korea; 2 Department of Child and Adolescent Counseling Psychology, Hanseo University, Seosan City, Republic of Korea

**Keywords:** bicultural acceptance attitude, depression, friend support, life satisfaction, moderated mediation model, multicultural adolescents, self-esteem

## Abstract

This study examines whether depression is associated with variations in the associations between bicultural acceptance attitudes and life satisfaction alongside friend support and self-esteem among adolescents from multicultural families with a Korean father and a foreign mother in South Korea. Based on a theoretically derived model, the study tests a moderated mediation framework using data from the fifth wave (2023) of the Multicultural Adolescents Panel Study (MAPS Phase 2). The analytic sample included 1,402 adolescents from families with Korean fathers and foreign mothers. Statistical analyses were conducted using SPSS 25.0, AMOS 23.0, and PROCESS macro (Model 91). The results indicate that bicultural acceptance attitudes are positively associated with friend support and self-esteem, which in turn are associated with life satisfaction. Furthermore, depression was associated with differences in the strength of the relationship between friend support and self-esteem, such that the association linking bicultural acceptance attitudes to life satisfaction alongside friend support became weaker at higher levels of depression. These findings are consistent with a moderated mediation pattern and suggest that depression is associated with variations in the pattern of the association among these psychosocial variables. The study provides theoretical and practical implications for supporting wellbeing among adolescents from multicultural families with a Korean father and a foreign mother.

## 1 Introduction

With Korean society increasingly transitioning toward multiculturalism, greater social attention and institutional efforts are required to support adolescents from multicultural families in adapting successfully and leading fulfilling lives (Ahn et al., 2024). Although adolescents’ life satisfaction encompasses multiple domains—including self, peer, school, and community contexts—the life satisfaction of multicultural adolescents warrants particular attention given their bicultural developmental context (Lee et al., 2021). Previous research has suggested that higher levels of bicultural acceptance are associated with enhanced life satisfaction (Park and Bae, 2024). However, the underlying psychosocial associations linking bicultural acceptance attitudes to life satisfaction remain insufficiently examined.

Emerging evidence suggests that friend support may play an important role in this association. Bicultural acceptance attitudes have been positively associated with adolescents’ perceived friend support (Lim, 2023), and friend support has been linked to life satisfaction alongside adaptive psychological processes such as resilience (Azpiazu Izaguirre et al., 2021). Building on these findings, the present study investigates whether friend support may be involved as a potential mediator in the association between bicultural acceptance attitudes and life satisfaction among multicultural adolescents.

Self-esteem represents another key psychological resource. Defined as an individual’s positive evaluation of the self (Muris and Otgaar, 2023), self-esteem has consistently been associated with adolescents’ adjustment and life satisfaction (Kim and Jung, 2024). Grounded in sociometer theory (Leary and Baumeister, 2000), self-esteem can be understood as an internal indicator of perceived social acceptance. From this perspective, friend support provides critical interpersonal feedback that informs adolescents’ self-evaluations, offering a theoretical basis for specifying a directional association from friend support to self-esteem. Prior research has also indicated that positive bicultural attitudes are associated with higher levels of self-esteem among multicultural youth (Kim and Kim, 2023). Nonetheless, the sequential interplay among bicultural acceptance, friend support, self-esteem, and life satisfaction has not been comprehensively examined.

Depression may further condition these relationships. Depressive symptoms have been negatively associated with social support and self-esteem (Ren et al., 2018; Masselink et al., 2018), and recent findings suggest that depression is associated with bicultural adaptation and life satisfaction (Choi and Um, 2024). Drawing on cognitive theories of depression (Beck, 1976), depressive symptoms are associated with negative cognitive biases that may be reflected in how individuals perceive and internalize social experiences. Accordingly, adolescents with higher levels of depression may be less likely to translate supportive peer interactions into positive self-evaluations. Rather than assuming a fixed process, it is therefore important to examine whether depression is associated with differences in the strength of the relationships linking bicultural acceptance attitudes to life satisfaction.

Accordingly, this study examines whether the association between bicultural acceptance attitudes and life satisfaction—alongside friend support and self-esteem—varies as a function of depression among multicultural adolescents. By investigating this conditional process model, the study seeks to provide evidence that may inform future longitudinal and intervention-based research aimed at supporting psychological wellbeing in multicultural youth.

This study aims to contribute to the existing literature in several ways. First, it seeks to integrate bicultural acceptance attitudes, interpersonal resources, and intrapersonal psychological associations into a unified sequential mediation framework. Second, it adopts a conditional process perspective, proposing that the associations between bicultural acceptance attitudes and life satisfaction may vary depending on adolescents’ psychological context, particularly levels of depression. Third, by focusing on multicultural adolescents, this study aims to provide context-specific insights into how cultural adaptation processes and mental health are jointly associated with wellbeing.

## 2 Theoretical background

### 2.1 Relationship between bicultural acceptance attitude and life satisfaction

Bicultural acceptance attitude has been conceptualized as multicultural adolescents’ positive orientation toward “simultaneously embracing both the heritage culture of their foreign parent and the mainstream culture” (Benet-Martínez and Haritatos, 2005). Choi and Um (2024) further describe bicultural acceptance as adolescents’ psychological readiness and flexibility to “engage with the tasks of both cultures.”

Life satisfaction, on the other hand, refers to an individual’s evaluation of the degree of satisfaction across various life domains, reflecting the combined role of internal and external factors (Diener and Fujita, 2005). For adolescents, a critical component of life satisfaction lies in satisfaction with friendships, as friendships fulfill essential needs for emotional support, intimacy, and self-worth recognition (Kaufman et al., 2022).

In the context of multicultural adolescents, prior research has examined the association between bicultural acceptance attitudes and life satisfaction. Empirical evidence suggests that positive bicultural acceptance is associated with better friendships, smoother school adjustment, and lower sensitivity to discrimination (Petkanopoulou et al., 2021).

However, multicultural adolescents often face acculturative stress when encountering a new cultural environment (Berry, 1992). Such stress may undermine their bicultural acceptance of both mainstream culture and their heritage culture (Nho and Hong, 2006), while negative bicultural attitudes have been found to be associated with lower levels of life satisfaction (Kim and Yoon, 2020).

Taken together, these findings suggest that bicultural acceptance attitudes are positively associated with life satisfaction. Accordingly, this study examines bicultural acceptance attitude and life satisfaction to further investigate the relationship between the two.

### 2.2 Double mediating roles of friend support and self-esteem

#### 2.2.1 Mediating role of friend support

Friend support, as one source of social support, refers to the care, respect, and sense of belonging that individuals derive from peer relationships (Cobb, 1976). It has also been defined as the extent to which adolescents receive recognition, advice, and tangible assistance from their friends (Demaray and Malecki, 2002).

A review of previous studies highlights two key strands of evidence. First, regarding the relationship between bicultural acceptance attitudes and friend support, research on multicultural adolescents in South Korea has shown that higher levels of bicultural acceptance are associated with greater perceived friend support (Lim, 2023). Similarly, children and adolescents who adjust well to school and establish positive relationships with peers and teachers tend to report higher levels of bicultural acceptance (Rutland et al., 2012).

Second, regarding the relationship between friend support and life satisfaction, empirical findings indicate that friend support is positively associated with life satisfaction and is also related to various psychosocial processes, such as resilience, emotion regulation, and loneliness (Azpiazu Izaguirre et al., 2021). Positive friendships are associated with adolescents’ needs for intimacy, greater social support, and lower levels of peer victimization, and are in turn associated with higher levels of life satisfaction (Gini et al., 2024).

Based on these findings, bicultural acceptance attitudes are expected to be associated with higher levels of friend support, which in turn is associated with life satisfaction. Accordingly, the present study hypothesizes that friend support mediates the association between bicultural acceptance attitudes and life satisfaction and seeks to examine this mediating relationship.

#### 2.2.2 Mediating role of self-esteem

Self-esteem is a psychological construct that reflects how individuals perceive and evaluate themselves, encompassing both positive self-acceptance and judgments of personal worth (Rosenberg, 1965). It has been regarded as a key factor influencing emotional stability and social functioning and is defined as the integration of competence and self-worth (Mruk, 2006).

Bicultural acceptance attitudes are closely linked to self-esteem. Multicultural adolescents must navigate the cultural expectations of both parents, and the resulting cultural conflicts and identity confusion may complicate the development of self-esteem (Lilgendahl et al., 2018). Empirical evidence has demonstrated that fostering bicultural acceptance attitudes can enhance self-esteem among multicultural adolescents (Um, 2024).

Self-esteem is also strongly associated with life satisfaction. Research indicates that high self-esteem provides immigrant adolescents with the psychological resources to cope with adverse environments, enabling them to maintain greater emotional stability, establish relationships, and communicate effectively with others (Harris and Orth, 2020). Multiple studies have further shown that self-esteem helps prevent problematic behaviors such as social withdrawal, thereby contributing to greater life satisfaction (Lee and Chang, 2020; Kim and Yoon, 2020).

Therefore, self-esteem may serve as an important mediator in the relationship between bicultural acceptance attitudes and life satisfaction among multicultural adolescents. Prior studies have confirmed that bicultural acceptance attitudes are associated with higher levels of self-esteem, which in turn increases life satisfaction (Jang et al., 2024). This suggests that self-esteem is not merely an emotional outcome variable but also functions as a psychosocial mediator linking bicultural acceptance attitudes and life satisfaction.

#### 2.2.3 Association between friend support and self-esteem

A growing body of research has demonstrated a significant positive association between friend support and self-esteem. For example, a study of Israeli adolescents found that friend support played a compensatory role for self-esteem in situations where maternal support was low (Hoffman et al., 1988). Similarly, in a pre-adolescent sample in Turkey, both friend support and family support significantly predicted self-esteem, underscoring the importance of friend support as a crucial source of social support (Kahyaoğlu, 2010).

Furthermore, recent evidence has shown that friend support indirectly reduces internalizing and externalizing psychological problems among college students alongside the enhancement of self-esteem. In other words, friend support is positively associated with both self-esteem and mental health (Szkody and McKinney, 2019).

Taken together, these findings suggest that friend support is considered a critical interpersonal resource that is closely linked to the development of self-esteem. From a social support perspective, external validation and a sense of belonging derived from peer relationships are associated with more positive self-evaluations. Therefore, in the present study, friend support is conceptually positioned in relation to self-esteem.

### 2.3 Moderating role of depression

Depression is an emotional disorder characterized by markedly low mood, lack of energy, and psychomotor retardation (Smith and de Torres, 2014). More specifically, depression is a psychological condition associated with difficulties across a wide range of domains, including perception, judgment, cognition, thinking, attitudes, and interpersonal relationships (Beck, 1976). Depression and anxiety are among the most common psychological problems that may emerge during adolescence (Huang et al., 2023).

The present study examines whether depression is associated with differences in the strength of the relationship between friend support and self-esteem, and whether depression is associated with variations in the relationships involving friend support and self-esteem in the association between bicultural acceptance attitudes and life satisfaction. Prior findings suggest that high levels of friend support are associated with weaker negative associations of risk factors such as acculturative stress or depressive symptoms (Um, 2024). Conversely, a longitudinal study of adolescents aged 11–17 found that depression was negatively associated with friend support, indicating that higher levels of depressive symptoms are linked to lower levels of friend support (Ren et al., 2018). In addition, numerous studies have reported a reciprocal association between adolescents’ self-esteem and depressive symptoms, with lower self-esteem being associated with greater severity of depression (Masselink et al., 2018). Clinical evidence further suggests that improvements in depressive symptoms following psychotherapeutic intervention are accompanied by increases in self-esteem (Bhattacharya et al., 2023).

Further evidence supports the role of depression as a moderating variable. For instance, a study of low-income children found that the positive association between physical activity and quality of life varied depending on levels of depression (Ra and Gang, 2016). Similarly, a study conducted among Korean middle school students showed that individuals who spent ≥1.5 h per day playing online games and reported higher levels of depression were more likely to engage in problematic online gaming. In other words, depression was associated with a stronger relationship between extended gaming time and negative outcomes (Jo et al., 2022).

Although prior research has examined the moderating role of depression, no study to date has investigated whether depression is associated with differences in the strength of the association between bicultural acceptance attitudes and life satisfaction via friend support and self-esteem. Thus, the present study seeks to address this gap by examining a moderated mediation pattern involving depression among adolescents from multicultural families in South Korea.

Importantly, depression may be associated with differences in the extent to which adolescents relate social support to positive self-evaluations. Given that depressive symptoms are associated with negative cognitive biases and reduced responsiveness to positive interpersonal experiences, individuals with higher levels of depression may be less likely to report stronger associations between friend support and self-esteem. Based on this perspective, the present study considers depression as a variable associated with differences in the relationship between friend support and self-esteem.

### 2.4 Hypotheses

Drawing on the theoretical framework and empirical literature discussed above, this study proposes a moderated mediation model linking bicultural acceptance attitude, friend support, self-esteem, depression, and life satisfaction among multicultural adolescents in South Korea. Specifically, bicultural acceptance attitude is expected to be associated with life satisfaction alongside the sequential mediating roles of friend support and self-esteem, while depression is expected to be associated with differences in this indirect pathway.

*H1*: Bicultural acceptance attitude is positively associated with friend support, self-esteem, and life satisfaction, and negatively associated with depression.

*H2*: Depression is associated with variations in the association between bicultural acceptance attitude and life satisfaction via friend support and self-esteem.

## 3 Research methods

### 3.1 Research model

**Figure 1:**
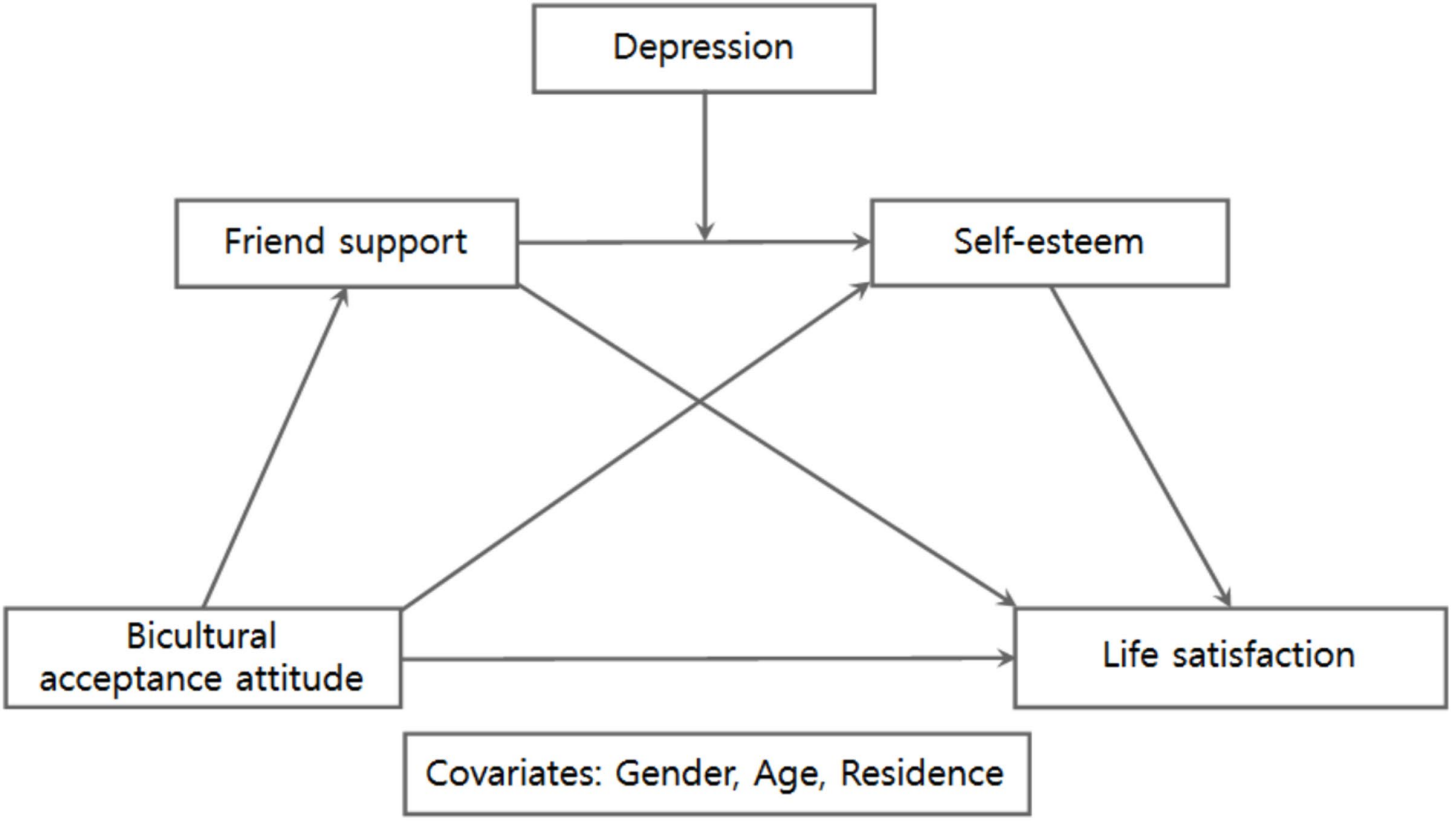
Conceptual research model.

This study examined whether depression is associated with differences in the indirect associations linking bicultural acceptance attitudes to life satisfaction via friend support and self-esteem among multicultural adolescents in Korea. The analysis was conducted using Model 91 of the PROCESS macro (version 4.2) developed by Hayes (2017). As shown in Figure 1, gender, age, and residential area were included as covariates, given their potential relevance to both the mediators and the outcome variable.

### 3.2 Participants and data collection

This study employed a cross-sectional analysis of data from the fifth wave (2023) of the Multicultural Adolescents Panel Study Phase 2 (MAPS Phase 2) conducted by the Korea Youth Policy Institute. MAPS Phase 2 is a nationally representative longitudinal panel survey designed to examine the developmental trajectories, psychosocial adjustment, and life outcomes of multicultural youth in South Korea. The present study utilized publicly available secondary data.

The target population consisted of multicultural youth enrolled in the fourth grade of elementary school nationwide in 2019. The sampling frame was constructed using national educational statistics provided by Korean Ministry of Education (2019), focusing specifically on multicultural fourth-grade students.

A stratified sampling method was used to select schools across geographic regions. Within the selected schools, a census survey was conducted among all eligible multicultural students. In 2019 and 2020, an initial panel of 2,249 households was established, including children from international marriage families, immigrant-background families, and foreign-national families.

For the present study, data were drawn from households that participated in the 2023 wave. Of the 1,877 households that participated in the 2023 wave, the present study focused on 1,402 cases involving families with a Korean father and a foreign mother. This restriction was applied to ensure greater sample homogeneity and comparability, as variations in parental cultural background and family structure may introduce additional heterogeneity that could confound the relationships examined in this study.

Data were collected between June and October 2023 through face-to-face household interviews. Structured questionnaires were administered using Tablet-Assisted Personal Interviewing (TAPI), which ensured standardized administration and minimized data entry errors. Trained interviewers conducted in-home visits following standardized survey protocols.

The study was approved for exemption from review by the Institutional Review Board (IRB) regarding the use of secondary data and was conducted in accordance with relevant ethical guidelines (Approval No. HS25-11-01).

The final sample consisted of 1,402 adolescents (52.5% male, 47.5% female). The mean age was 14.02 years (range = 13–17), with the majority aged 14 years (96.6%). Regarding residential area size, 51.3% resided in small- or medium-sized cities, 30.4% in metropolitan cities, and 18.3% in rural areas.

### 3.3 Measurement instruments

#### 3.3.1 Bicultural acceptance attitude

To assess bicultural acceptance attitudes among youth from multicultural families, the present study utilized the scale developed by Nho and Hong (2006). The original instrument comprised 10 items, each rated on a 4-point Likert scale (1 = Not at all, 4 = Very much), with higher scores indicating a greater degree of bicultural acceptance attitude.

To examine the latent structure of bicultural acceptance, an exploratory factor analysis (EFA) using principal axis factoring with Promax rotation was initially conducted on all 10 items. The Kaiser–Meyer–Olkin (KMO) value was 0.798, and Bartlett’s test of sphericity was significant (χ^2^ = 5248.763, *p* < 0.001), indicating the suitability of the data for factor analysis. The initial solution explained 49.74% of the total variance; however, four items (Items 1, 2, 3, and 9) showed low communalities (below 0.40) and weak contributions to the factor structure and were therefore excluded. For transparency, examples of deleted items include: “I tend to enjoy my mother’s native culture (e.g., music, movies, food, clothing)” and “I tend to enjoy Korean culture (e.g., music, movies, food, clothing).” These items reflected parallel aspects of bicultural orientation but did not load clearly on the intended factor, suggesting limited alignment with the underlying construct of bicultural acceptance in this sample context.

A subsequent EFA was conducted with the remaining six items. The KMO value was 0.692, and Bartlett’s test of sphericity remained significant (χ^2^ = 2823.192, *p* < 0.001), supporting the adequacy of the data. The two-factor solution explained 58.05% of the total variance and yielded a clearer and theoretically interpretable factor structure. Overall, while the general factor structure remained consistent, the removal of the four items improved the clarity and robustness of the measurement.

Given that the structural model was based on a single overarching construct, a second-order confirmatory factor analysis (CFA) was performed. The higher-order factor loaded significantly onto both first-order factors, supporting a hierarchical structure. After allowing theoretically justified error covariances, the modified model demonstrated acceptable fit (χ^2^ = 37.509, df = 5, χ^2^/df = 7.502, GFI = 0.991, CFI = 0.988, TLI = 0.965, RMSEA = 0.068).

It should be noted that both exploratory and confirmatory factor analyses were conducted using the same dataset due to data constraints. Accordingly, the stability of the factor structure should be interpreted with caution, and future research is needed to validate the measurement model using independent samples.

Convergent validity and reliability were assessed at the first-order level. AVE values were 0.504 and 0.714, exceeding the 0.50 threshold, and composite reliability (CR) values were 0.800 and 0.829, respectively. Although Cronbach’s alpha was attenuated due to the limited number of items, McDonald’s omega coefficients (0.800 and 0.829) indicated satisfactory internal consistency.

Discriminant validity was supported using both the Fornell–Larcker criterion and the heterotrait–monotrait ratio (HTMT). The square roots of AVE exceeded inter-factor correlations, and all HTMT values were below the conservative threshold of 0.85 (Henseler et al., 2015), confirming the empirical distinctiveness of the first-order factors.

Overall, the findings suggest that bicultural acceptance can be represented as a reliable and valid hierarchical two-factor structure, supporting the use of a higher-order latent construct in subsequent analyses.

#### 3.3.2 Friend support

Friend support was measured using a revised version of the scale originally developed by Han (1996), tailored to the context of the Multicultural Adolescents Panel Study (MAPS) dataset. The scale comprises three items designed to capture key behavioral expressions of social support among peers. Respondents rated each item on a 5-point Likert scale ranging from 1 (“Not at all true”) to 5 (“Very true”), with higher scores indicating a greater perceived level of support from friends.

An exploratory factor analysis (EFA) using principal axis factoring with Promax rotation was conducted to examine the latent structure of friend support. The Kaiser–Meyer–Olkin (KMO) measure was 0.744, indicating acceptable sampling adequacy (Kaiser, 1974), and Bartlett’s test of sphericity was significant (χ^2^ = 2489.466, *p* < 0.001), confirming the suitability of the data for factor analysis. A single-factor solution emerged, accounting for 73.70% of the total variance. All three items loaded strongly on the factor (*λ* > 0.80), supporting a clear unidimensional structure.

Convergent validity and construct reliability were further examined using confirmatory factor analysis (CFA). Convergent validity and reliability were satisfactory. The average variance extracted (AVE) was 0.737 and composite reliability (CR) was 0.894, exceeding recommended thresholds (AVE ≥ 0.50; CR ≥ 0.70) (Fornell and Larcker, 1981; Hair et al., 2019). Cronbach’s alpha (0.893) and McDonald’s omega (0.894) further indicated strong internal consistency.

Although the scale consists of three items, it meets the minimum requirement for latent variable identification in structural equation modeling (Bollen, 1989; Kline, 2016). Given the high factor loadings, substantial explained variance, and strong reliability indices, the three-item measure demonstrates adequate psychometric robustness. Prior research suggests that brief scales can perform reliably when they clearly represent a unidimensional construct with strong loadings (Worthington and Whittaker, 2006; Gosling et al., 2003).

Overall, the friend support scale exhibits satisfactory validity and reliability for use in subsequent structural analyses.

#### 3.3.3 Depression

Depression was assessed using a revised version of the instrument originally developed by Ha et al. (2018), adapted for adolescent populations. The scale consists of five items measuring depressive mood states, each rated on a 4-point Likert scale ranging from 1 (“Not at all true”) to 4 (“Very true”), with higher scores indicating greater depressive symptoms.

To examine the latent structure of depression, an exploratory factor analysis (EFA) was conducted using principal axis factoring with Promax rotation. Principal axis factoring was selected as it is appropriate for common factor models that assume latent constructs, and oblique rotation was applied to account for potential inter-item correlations.

The Kaiser–Meyer–Olkin (KMO) measure was 0.843, indicating good sampling adequacy (Kaiser, 1974), and Bartlett’s test of sphericity was significant (χ^2^ = 3864.645, *p* < 0.001), confirming the suitability of the data for factor analysis. A single-factor solution emerged, explaining 61.442% of the total variance. All items loaded strongly on the factor (*λ* ≥ 0.70), supporting a theoretically interpretable unidimensional structure. The substantial explained variance further indicates that the items adequately reflect a common underlying construct.

Convergent validity and construct reliability were subsequently examined using confirmatory factor analysis (CFA). The average variance extracted (AVE) was 0.607 and composite reliability (CR) was 0.885, exceeding recommended thresholds (AVE ≥ 0.50; CR ≥ 0.70) (Fornell and Larcker, 1981; Hair et al., 2019). Cronbach’s alpha (0.885) and McDonald’s omega (0.885) also indicated strong internal consistency (McDonald, 1999). The AVE value approaching 0.70 suggests that a substantial proportion of item variance is explained by the latent construct.

Although the scale comprises five items, it exceeds the minimum requirement for latent variable identification in structural equation modeling (Bollen, 1989; Kline, 2016). Given the strong factor loadings, substantial explained variance, and robust reliability indices, the depression measure demonstrates adequate psychometric soundness for use in subsequent structural analyses. Brief scales with clearly defined unidimensional structures have been shown to function reliably in structural models when supported by strong empirical evidence (Worthington and Whittaker, 2006; Gosling et al., 2003).

Accordingly, the depression scale used in this study can be considered psychometrically valid and reliable.

#### 3.3.4 Self-esteem

Self-esteem was measured using three modified items derived from the Comprehensive Adolescent Survey (Baek et al., 2017). Each item was rated on a 4-point Likert scale ranging from 1 (“Not at all true”) to 4 (“Very true”), with higher scores indicating higher levels of self-esteem.

To examine the latent structure of self-esteem, an exploratory factor analysis (EFA) was conducted using principal axis factoring with Promax rotation. Principal axis factoring was chosen as it is appropriate for common factor models assuming latent constructs, and oblique rotation was applied to account for potential inter-item correlations.

The Kaiser–Meyer–Olkin (KMO) measure was 0.741, indicating acceptable sampling adequacy (Kaiser, 1974), and Bartlett’s test of sphericity was significant (χ^2^ = 2195.219, *p* < 0.001), confirming the suitability of the data for factor analysis. A single-factor solution emerged, explaining 70.544% of the total variance. All items loaded strongly on the factor (*λ* ≥ 0.80), supporting a theoretically interpretable unidimensional structure. The substantial explained variance indicates that the items robustly reflect a common latent construct.

Convergent validity and construct reliability were further examined using confirmatory factor analysis. The average variance extracted (AVE) was 0.706 and composite reliability (CR) was 0.878, exceeding recommended thresholds (AVE ≥ 0.50; CR ≥ 0.70) (Fornell and Larcker, 1981; Hair et al., 2019). Cronbach’s alpha (0.876) and McDonald’s omega (0.878) also demonstrated strong internal consistency (McDonald, 1999). The AVE value approaching 0.70 suggests that a substantial proportion of item variance is explained by the latent factor.

Although the scale comprises three items, it meets the minimum requirement for latent variable identification in structural equation modeling (Bollen, 1989; Kline, 2016). Given the high factor loadings, substantial explained variance, and robust reliability indices, the three-item measure demonstrates adequate psychometric soundness. Prior research suggests that brief unidimensional scales with strong loadings can function reliably in structural models (Worthington and Whittaker, 2006; Gosling et al., 2003).

Accordingly, the self-esteem scale used in this study can be considered both valid and reliable for subsequent structural analyses.

#### 3.3.5 Life satisfaction

Life satisfaction was measured using a three-item scale originally developed by Kim et al. (2010) and adapted for use with the Multicultural Adolescents Panel Study (MAPS). Each item was rated on a 4-point Likert scale ranging from 1 (“Not at all true”) to 4 (“Very true”), with higher scores indicating greater life satisfaction.

To examine the latent structure, an exploratory factor analysis (EFA) was conducted using principal axis factoring with Promax rotation. The Kaiser–Meyer–Olkin (KMO) measure was 0.691, indicating acceptable sampling adequacy (Kaiser, 1974), and Bartlett’s test of sphericity was significant (χ^2^ = 2048.662, *p* < 0.001), confirming the appropriateness of factor analysis. A single-factor solution emerged, explaining 67.36% of the total variance. All items loaded strongly on the factor, supporting a clear unidimensional structure. The substantial explained variance indicates adequate convergence despite the brevity of the scale.

Convergent validity and construct reliability were further examined using confirmatory factor analysis (CFA). The average variance extracted (AVE) was 0.674 and composite reliability (CR) was 0.859, exceeding recommended thresholds (AVE ≥ 0.50; CR ≥ 0.70) (Fornell and Larcker, 1981; Hair et al., 2019). Cronbach’s alpha (0.833) and McDonald’s omega (0.859) also indicated satisfactory internal consistency (McDonald, 1999). The AVE value above 0.60 suggests that a substantial proportion of item variance is explained by the latent construct.

Although the scale comprises three items, it satisfies the minimum requirement for latent variable identification in structural equation modeling (Bollen, 1989; Kline, 2016). Given the strong factor loadings, substantial explained variance, and robust reliability indices, the measure demonstrates adequate psychometric soundness for subsequent structural analyses. Brief unidimensional scales have been shown to perform reliably when supported by strong empirical evidence (Worthington and Whittaker, 2006; Gosling et al., 2003).

Overall, the life satisfaction scale used in this study can be considered both valid and reliable.

#### 3.3.6 Control variables

In this study, sociodemographic characteristics such as gender, age, and residence were included as control variables to account for their associations with the mediating and dependent variables.

### 3.4 Common method Bias test

To assess the potential threat of common method bias (CMB), both procedural and statistical remedies were implemented. Procedurally, respondent anonymity was ensured, and items across constructs were presented in varied order to reduce evaluation apprehension and consistency motives, which are recommended strategies for minimizing method bias.

As an initial diagnostic, Harman’s single-factor test was conducted by entering all measurement items into an exploratory factor analysis without rotation. The first factor accounted for 34.34% of the total variance, which is well below the 50% threshold suggested by Podsakoff et al. (2003). This result indicates that a single factor does not dominate the covariance structure and suggests that common method bias is unlikely to be a serious concern.

In addition, a Common Latent Factor (CLF) was incorporated into the measurement model to account for potential common method variance (CMV). The inclusion of the CLF resulted in modest improvements in model fit (CMIN/DF = 3.701, CFI = 0.979, TLI = 0.970, RMSEA = 0.044) compared to the baseline model (CMIN/DF = 4.869, CFI = 0.965, TLI = 0.957, RMSEA = 0.053). Importantly, the standardized factor loadings and substantive structural path coefficients remained substantively unchanged after controlling for the CLF. Differences in loadings were minimal and did not alter the magnitude, direction, or statistical significance of the hypothesized relationships.

Furthermore, the proportion of variance attributable to the CLF was substantially smaller than that explained by the substantive latent constructs, indicating that the majority of variance is driven by theoretically meaningful factors rather than method effects.

Taken together, these findings suggest that common method bias may not be a dominant source of variance in this study. However, given the reliance on self-reported data collected at a single time point, the possibility of common method bias cannot be fully ruled out. Accordingly, the results should be interpreted with caution.

### 3.5 Data analysis

Data analysis was conducted using IBM SPSS Statistics 25.0, AMOS 23.0, and the PROCESS macro (version 4.2). A two-step analytical strategy was adopted to ensure both measurement validity and robust hypothesis testing.

First, the measurement model was evaluated. Exploratory factor analysis (EFA) and confirmatory factor analysis (CFA) were conducted to assess construct validity, while internal consistency reliability was examined using Cronbach’s alpha and McDonald’s omega coefficients. AMOS was specifically used to validate the measurement structure and confirm the adequacy of the latent constructs.

Second, the hypothesized relationships were tested using the PROCESS macro (Hayes, 2018). PROCESS is well suited for estimating conditional associations and indirect effects within regression-based models using bootstrapping. In this study, moderated mediation effects were examined using Model 91. A bootstrapping procedure with 5,000 resamples was employed to generate bias-corrected 95% confidence intervals (CIs). Conditional indirect effects were estimated at low (−1 SD), mean, and high (+1 SD) levels of the moderator. Statistical significance was determined when the 95% CI did not include zero. Although latent constructs were validated using CFA, the hypothesized relationships were tested using observed variables within the PROCESS framework. Accordingly, measurement error was not explicitly modeled in the structural analysis.

In addition, descriptive statistics and frequency analyses were conducted to examine participants’ general characteristics, and Pearson’s correlation analysis was used to assess relationships among key variables. To address potential common method bias, both Harman’s single-factor test and the common latent factor (CLF) approach were applied.

## 4 Research results

### 4.1 Correlation among variables

Pearson correlation analyses revealed that all study variables were significantly associated (*p* < 0.01). Depression was negatively correlated with bicultural acceptance attitudes (*r* = −0.249), friend support (*r* = −0.284), self-esteem (*r* = −0.470), and life satisfaction (*r* = −0.519). The remaining variables were positively intercorrelated, with a strong association between self-esteem and life satisfaction (*r* = 0.754). These results are consistent with the expected associations (H1).

Although the correlation between self-esteem and life satisfaction was relatively high, the constructs satisfied established criteria for discriminant validity (e.g., AVE), indicating that they represent related but distinct psychological constructs.

All intercorrelations among independent variables were below *r* = 0.70, indicating no multicollinearity concerns (Kline, 2011; Hair et al., 2019).

Descriptive statistics showed relatively low levels of depression (*M* = 1.56, SD = 0.57; 4-point scale) and relatively high levels of friend support (*M* = 3.94, SD = 0.66; 5-point scale), self-esteem (*M* = 3.21, SD = 0.56), and life satisfaction (*M* = 3.12, SD = 0.58; 4-point scales). Skewness (−0.306 to 0.988) and kurtosis (0.095–0.929) values were within acceptable ranges, supporting the assumption of normality (Kline, 2011; Table 1).

**Table 1:** Descriptive statistics and correlations among study variables.

Categories	1	2	3	4	5
1. Bicultural acceptance attitude	1				
2. Friend support	0.334^**^	1			
3. Depression	−0.249^**^	−0.284^**^	1		
4. Self-esteem	0.359^**^	0.425^**^	−0.470^**^	1	
5. Life satisfaction	0.278^**^	0.412^**^	−0.519^**^	0.754^**^	1
M	3.2502	3.9391	1.5572	3.2061	3.1217
SD	0.47329	0.66173	0.57081	0.55692	0.58337
Skewness	−0.306	−0.277	0.988	−0.214	−0.144
Kurtosis	0.894	0.140	0.929	0.361	0.095

**p < 0.01.

### 4.2 Moderated dual mediation model of depression

**Figure 2:**
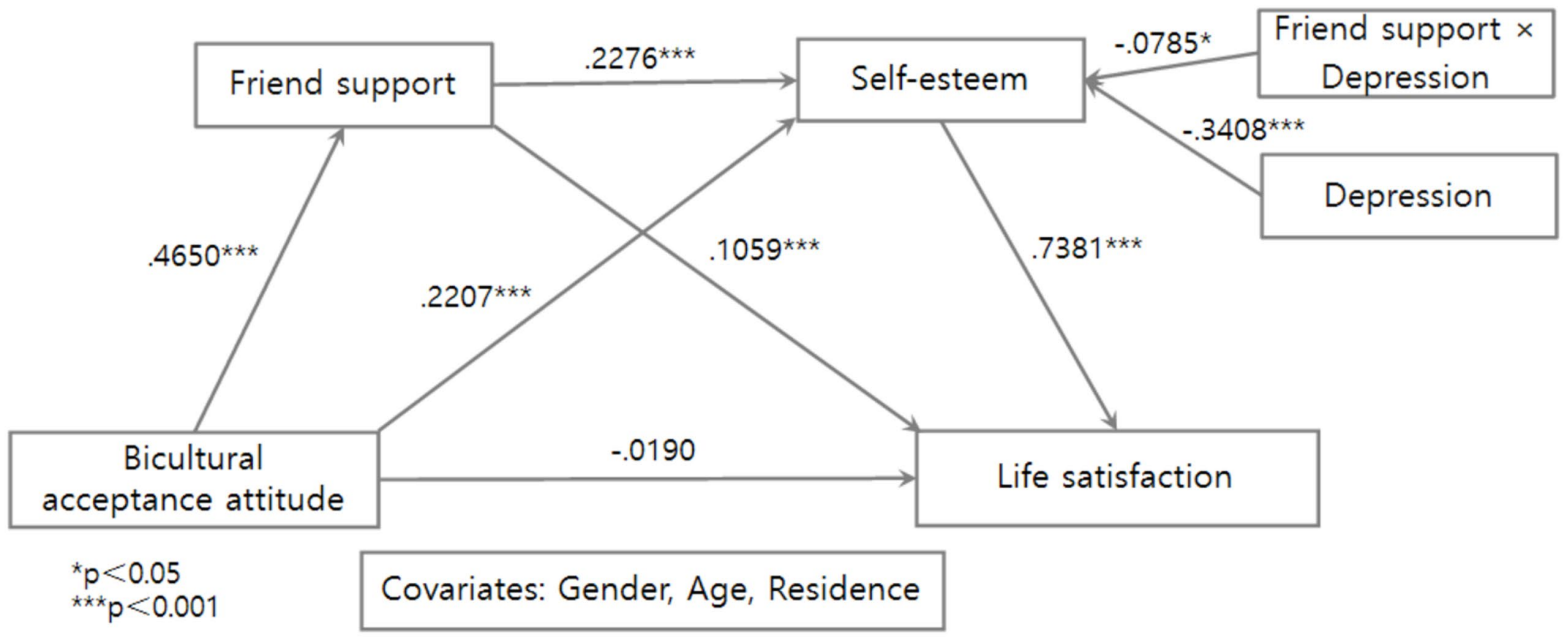
Statistical model of moderated mediation involving depression.

**Figure 3:**
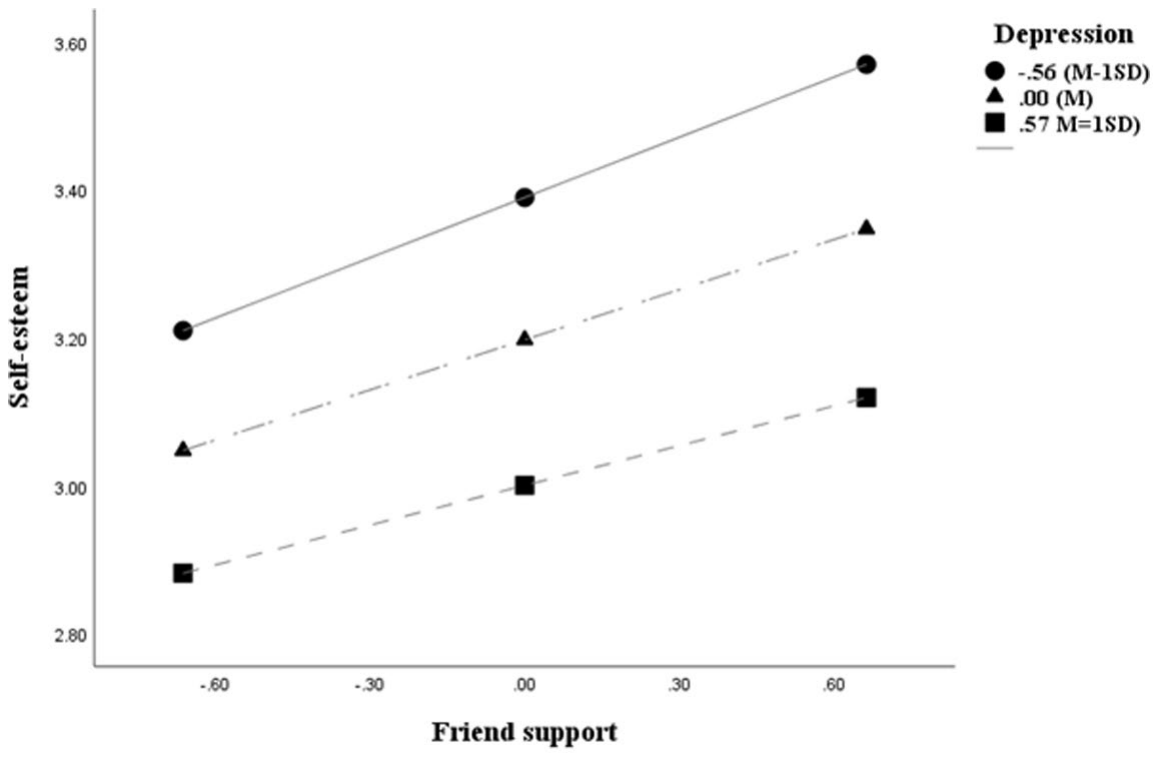
Moderating role of depression.

Moderated mediation was tested using PROCESS Model 91 with 5,000 bootstrap resamples while controlling for gender, age, and residence. The analysis results are shown in Figures 2, 3 and Tables 2, 3.

**Table 2:** Moderating role of depression.

Variable		Mediating variable model 1 (DV: friend support)			Mediating variable mode 2 (DV: self-esteem)			Dependent variable model (DV: life satisfaction)		
		Coeffect	SE	t value	Coeffect	SE	t value	Coeffect	SE	t value
Constant		−1.5556	1.1060	−1.4065	2.2064	0.7977	2.7661^**^	2.5545	0.6714	3.8049^***^
IV	Bicultural acceptance attitude	0.4650	0.0352	13.2064^***^	0.2207	0.0273	8.0800^***^	−0.0190	0.0234	−0.8116
Mediator1	Friend support				0.2276	0.0198	11.5104^***^	0.1059	0.0173	6.1129^***^
Mediator2	Self-esteem							0.7381	0.0209	35.3209^***^
Moderator	Depression				−0.3408	0.0227	−15.0147^***^			
Interaction item	Friend support × Depression				−0.0785	0.0309	−2.5403^*^			
Highest order test	R^2^ change				0.0030					
	F				6.4531^*^					
Covariates	Gender	0.0614	0.0334	1.8403	−0.0924	0.0242	−3.8165^***^	−0.0566	0.0204	−2.7732^**^
	Age	−0.0074	0.0779	−0.0955	0.0276	0.0561	0.4929	−0.1159	0.0472	−2.4559^*^
	Residence	0.0176	0.0131	1.3463	0.0069	0.0094	0.7318	−0.0090	0.0079	−1.1392
Model summary	$R^{2}$	0.1149			0.3533			0.5828		
	F	45.3285^***^			108.7943^***^			324.8329^***^		
Conditional effects of the friend support at values of the depression:										
Depression	Effect(B)		se		*t*-value		LLCI^*^		ULCI^**^	
−0.5572 (M-1SD)	0.2713		0.0262		10.3505^***^		0.2199		0.3227	
0.0000 (M)	0.2276		0.0198		11.5104^***^		0.1888		0.2664	
0.5708 (M + 1SD)	0.1828		0.0265		6.8973^***^		0.1308		0.2347	
Moderator value(s) defining Johnson-Neyman significance region(s):										
Value			% below				% above			
1.5817			98.7161				1.2839			
Conditional effect of friend support at values of the depression:										
Depression	Effect(B)		se		t value		LLCI^*^		ULCI^**^	
−0.5572	0.2713		0.0262		10.3505^***^		0.2199		0.3227	
. .										
1.5428	0.1065		0.0516		2.0630^*^		0.0052		0.2077	
**1.5817**	**0.1034**		**0.0527**		**1.9617**		**0.0000**		**0.2069**	
1.6928	0.0947		0.0559		1.6935		−0.0150		0.2044	
. .										
2.4428	0.0358		0.0780		0.6461		−0.1172		0.1889	

^*^p < 0.05, ^**^p < 0.01, ^***^p < 0.001. *LLCI = Lower limit of the 95% bootstrap confidence interval. **ULCI = Upper limit of the 95% bootstrap confidence interval.

Moderator values are shown in bold.

**Table 3:** Direct and indirect patterns of bicultural acceptance attitude on life satisfaction.

Direct association (bicultural acceptance attitude → Life satisfaction)				
Effect	se	t value	LLCI^*^	ULCI^**^
−0.0190	0.0234	−0.8116	−0.0649	0.0269
Conditional indirect association (Bicultural acceptance attitude → Friend support → Self-esteem → Life satisfaction)				
Depression	Effect	BootSE	BootLLCI	BootULCI
−0.5572 (M-1SD)	0.0931	0.0125	0.0702	0.1194
0.0000 (M)	0.0781	0.0105	0.0582	0.0995
0.5708 (M + 1SD)	0.0627	0.0148	0.0337	0.0917

*LLCI = lower limit of the 95% bootstrap confidence interval. **ULCI = upper limit of the 95% bootstrap confidence interval.

Bicultural acceptance attitude was positively associated with friend support (*B* = 0.4650, *p* < 0.001) and self-esteem (*B* = 0.2207, *p* < 0.001), but its direct association with life satisfaction was not significant (*B* = −0.0190, ns). Friend support was positively associated with self-esteem (*B* = 0.2276, *p* < 0.001) and life satisfaction (*B* = 0.1059, *p* < 0.001), and self-esteem was strongly associated with life satisfaction (*B* = 0.7381, *p* < 0.001).

Depression is negatively associated with self-esteem (*B* = −0.3408, *p* < 0.001). The interaction between friend support and depression was significant (*B* = −0.0785, *p* < 0.05; Δ*R*^2^ = 0.0030), indicating that depression was associated with differences in the association between friend support and self-esteem. Specifically, the positive association between friend support and self-esteem decreased as depression increased.

Conditional effects analysis showed that the association between friend support and self-esteem remained significant at low (*B* = 0.2713), mean (*B* = 0.2276), and high (*B* = 0.1828) levels of depression (all *p* < 0.001), but the magnitude progressively weakened. This pattern suggests that depression is associated with weaker, but does not eliminate, the positive relationship between friend support and self-esteem.

Johnson–Neyman analysis indicated that the association became nonsignificant when depression exceeded 1.5817 (1.28% of the sample), suggesting that the positive association between friend support and self-esteem is stronger at lower to moderate levels of depression.

The conditional associations between friend support and self-esteem at three levels of depression (M − 1 SD, M, and M + 1 SD) are presented in Figure 3. The positive association between friend support and self-esteem was strongest at low levels of depression (M − 1 SD), weaker at the mean level, and weakest at high levels of depression (M + 1 SD). Although the relationship remained statistically significant across all levels, the slope decreased as depression increased, suggesting that higher levels of depression are associated with a weaker positive relationship between friend support and self-esteem.

Conditional indirect effects (i.e., conditional associations) were estimated to examine whether depression was associated with differences in the relationships linking bicultural acceptance attitude to life satisfaction via friend support and self-esteem (PROCESS Model 91, 5,000 bootstrap resamples).

The direct association between bicultural acceptance attitude and life satisfaction was not significant [*B* = −0.0190, 95% CI (−0.0649, 0.0269)]. In contrast, the sequential indirect association with friend support and self-esteem was significant at all three levels of depression, as the 95% bootstrap confidence intervals did not include zero. Specifically, the indirect association was strongest at low levels of depression [*M* − 1 SD; B = 0.0931, 95% CI (0.0702, 0.1194)], weaker at the mean level [*B* = 0.0781, 95% CI (0.0582, 0.0995)], and weakest at high levels of depression [M + 1 SD; *B* = 0.0627, 95% CI (0.0337, 0.0917)].

These findings indicate significant moderated mediation. Although bicultural acceptance attitude was not directly associated with life satisfaction, it was associated with life satisfaction alongside higher levels of friend support and self-esteem. However, the magnitude of this indirect association decreased as depression increased, suggesting that depression is associated with a weaker pattern of relationships linking bicultural acceptance attitude to life satisfaction. Therefore, Hypothesis 2 was supported.

## 5 Discussion and conclusion

First, the correlation analyses indicated that bicultural acceptance attitude was positively associated with friend support, self-esteem, and life satisfaction. These findings suggest that individuals who more readily accept and integrate bicultural identities tend to report stronger interpersonal resources and more positive self-evaluations, which in turn are linked to greater overall wellbeing. This pattern is consistent with prior research demonstrating that bicultural acceptance is positively related to social support and psychological adjustment (Jo and Kim, 2023). Specifically, Jo and Kim (2023) reported that social support—including friend support—and self-esteem was positively correlated with bicultural acceptance attitude and both were positively associated with higher levels of life satisfaction.

In contrast, depression was negatively correlated with bicultural acceptance attitude, friend support, self-esteem, and life satisfaction. These findings align with the broader literature indicating that depressive symptoms undermine both internal psychological resources and external social functioning. Higher levels of social support and self-esteem were associated with lower levels of depression, supporting the view that these variables function as protective factors against psychological distress (Liu et al., 2021). Social support may be associated with lower stress and more adaptive coping adaptive coping, whereas self-esteem enhances resilience by fostering a positive self-concept.

Furthermore, depression showed a strong negative association with life satisfaction, which is consistent with prior findings that depressive symptoms are associated with lower levels of subjective wellbeing (Choi and Um, 2024). Conversely, bicultural acceptance attitude appears to be associated with a protective pattern, being indirectly associated with life satisfaction alongside friend support and self-esteem.

However, the relatively strong association between self-esteem and life satisfaction warrants careful interpretation. Although these constructs are theoretically distinct, their conceptual proximity may be associated with the strength of the observed relationships. Therefore, the magnitude of the mediation pathway should be interpreted with caution.

Taken together, these findings suggest that bicultural acceptance attitude is associated with psychosocial adaptation, alongside interpersonal and intrapersonal resources, whereas depression is associated with multiple indicators of adjustment. The results highlight the importance of bicultural acceptance, supportive peer relationships, and self-esteem in understanding variations in life satisfaction, while also underscoring the need to consider depressive symptoms. These findings are consistent with the proposed hypotheses, supporting the hypothesized relationships among bicultural acceptance attitude, friend support, self-esteem, and life satisfaction.

Second, the moderated mediation analysis demonstrated that the indirect association of bicultural acceptance attitude with life satisfaction alongside friend support and self-esteem varied as a function of depression. Although the sequential indirect association was statistically significant at low (M − 1 SD), mean (M), and high (M + 1 SD) levels of depression, its magnitude decreased as depressive symptoms increased. This pattern suggests that the association between cultural adaptation and wellbeing becomes weaker—but does not disappear—at higher levels of depression. From a stress-buffering perspective (Cohen and Wills, 1985), friend support is associated with better psychological adjustment, and may be linked to processes such as emotional validation and instrumental resources, which are in turn associated with higher self-esteem. Bicultural acceptance attitude may be related to greater social integration and identity coherence, which is associated with stronger peer relationships and more positive self-evaluations. However, depressive symptoms are associated with cognitive distortions (e.g., negative self-schemas, reduced reward sensitivity), which may be related to lower levels of perceived social support. Thus, even when friend support is present, the association between friend support and self-esteem appears to be weaker among adolescents with higher levels of depression, suggesting a weaker overall pattern of relationships linking bicultural acceptance attitude to life satisfaction.

Several alternative interpretations should be considered. First, depression may be associated with lower levels of perceived—rather than actual—friend support, potentially reflecting cognitive bias rather than diminished social resources. Second, depressive withdrawal behaviors may be related to reduced engagement with supportive peers, which may in turn be associated with weaker relationships between support and psychological outcomes. Third, shared method variance or negative affectivity may be associated with the observed relationships between depression and other psychosocial variables. Longitudinal or multi-informant data would be needed to disentangle these possibilities.

The role of depression may vary depending on developmental stage, cultural context, and symptom severity. For example, the observed pattern of weaker associations may be more pronounced during adolescence, when peer relationships are central to identity formation. It may also be particularly evident in collectivistic cultural contexts, where social belonging carries heightened psychological significance. Importantly, the results indicate that the association linking bicultural acceptance to life satisfaction via friend support and self-esteem remains significant across most levels of depression, suggesting that bicultural acceptance may function as a resilience-related factor, particularly when depressive symptoms are not severe.

Because the association linking bicultural acceptance to life satisfaction via friend support and self-esteem becomes weaker—but does not disappear—as depression increases, the findings suggest that interventions might consider enhancing friend support. Rather, efforts to reduce depressive symptoms and address maladaptive cognitions may help adolescents more fully benefit from available social resources. Programs that simultaneously promote bicultural acceptance, strengthen peer networks, and support self-esteem may be more effective in supporting life satisfaction than single-component interventions.

The present findings extend prior work demonstrating that depression is associated with weaker positive associations between adaptive behaviors and quality of life (e.g., Ra and Gang, 2016). Whereas earlier studies primarily examined direct moderation roles, this study suggests that depression is associated with differences in the pattern of relationships linking cultural adaptation to wellbeing. Thus, the results are informative for integrative models of multicultural adaptation, indicating that internalizing symptoms are related to how cultural and relational resources are associated with subjective wellbeing.

While the present study extends prior research by incorporating a conditional process perspective, its contribution should be understood as an incremental step toward a more comprehensive understanding of multicultural adolescents’ psychological adjustment.

In sum, bicultural acceptance attitude is associated with life satisfaction alongside higher levels of friend support and self-esteem; however, this pattern of associations becomes weaker at higher levels of depression. These findings highlight the dynamic relationships among cultural adaptation, interpersonal resources, and internal psychological states, suggesting that mental health is not only associated with adjustment outcomes but also with variations in the strength of these associations among multicultural adolescents.

The present findings contribute to the existing literature in several ways. First, they integrate bicultural acceptance attitudes, interpersonal resources, and intrapersonal psychological associations into a unified sequential mediation framework. Second, they extend prior research by adopting a conditional process perspective, demonstrating that the associations between bicultural acceptance attitudes and life satisfaction vary depending on adolescents’ psychological context, particularly levels of depression, and by identifying a moderated mediation pattern in which depression is associated with differences in the strength of these relationships. Third, by focusing on multicultural adolescents, the findings provide context-specific insights into how cultural adaptation processes and mental health are jointly associated with wellbeing. Overall, these results are consistent with the hypothesized moderated mediation model.

Several limitations should be noted. First, the measurement of bicultural acceptance attitude was refined through the removal of four items with low communalities. Although this process improved the clarity and internal structure of the scale, it may have altered the original construct representation. Furthermore, because the measurement model was both developed and validated within the same dataset, the stability and generalizability of the refined scale remain uncertain. Therefore, the findings should be interpreted with caution, and future research is needed to cross-validate the factor structure using independent samples.

Second, the sample was restricted to adolescents from families with a Korean father and a foreign mother to ensure homogeneity. While this approach strengthens internal validity, it may limit the generalizability of the findings to other types of multicultural family structures with different parental compositions.

Third, the constructs of friend support and self-esteem were assessed using relatively brief measures. Although these measures demonstrated acceptable reliability, they may not fully capture the complexity and multidimensionality of the constructs. Future studies may benefit from employing more comprehensive scales to provide a deeper understanding of these relationships.

Fourth, exploratory and confirmatory factor analyses were conducted using the same dataset, which may raise concerns regarding the stability of the measurement model. Because cross-validation with an independent sample was not feasible, the factor structure should be interpreted with caution. Future research is needed to replicate and validate the measurement model using independent samples to ensure its robustness.

Fifth, although measurement validity was established using CFA, the main analyses were conducted using observed variables in a regression-based framework. Accordingly, measurement error was not explicitly modeled. In addition, the analytic strategy reflects a degree of inconsistency between latent construct validation and observed-variable hypothesis testing. While CFA was conducted to establish measurement validity, the use of observed variables in the PROCESS framework may limit the extent to which measurement error is accounted for. Accordingly, the findings should be interpreted with caution, particularly with respect to the structural relationships examined in this study. Future research may benefit from employing structural equation modeling to simultaneously account for measurement error and test the hypothesized relationships.

Sixth, as all variables were measured using self-reports at a single time point, common method bias may have influenced the observed associations. Although preliminary tests were conducted, this issue cannot be fully ruled out and should be considered when interpreting the findings.

Finally, the relatively high correlation between self-esteem and life satisfaction may raise concerns regarding their empirical distinctiveness. Although discriminant validity criteria were satisfied, the conceptual overlap between these constructs should be considered when interpreting the mediation results.

## Data Availability

Publicly available datasets were analyzed in this study. This data can be found at: https://www.nypi.re.kr/archive/mps.
